# Demographics, Services, and Practices in Attention-Deficit/Hyperactivity Disorder Coaching in the US

**DOI:** 10.1001/jamanetworkopen.2025.52407

**Published:** 2026-01-15

**Authors:** Margaret H. Sibley, Elias D. Graham, Jillian K. Holbrook, Melissa R. Dvorsky, Carlos E. Yeguez, Tamara Rosier, David Coghill, Timothy F. Page, Roxanne Fouche, Jami Demuth

**Affiliations:** 1 Department of Psychiatry and Behavioral Sciences, University of Washington School of Medicine, Seattle; 2 Center for Child Health, Behavior, and Development, Seattle Children’s Research Institute, Seattle, Washington; 3 Department of Psychology, University of Washington, Seattle; 4 Children’s National Research Institute, Washington, DC; 5 Department of Psychiatry & Behavioral Sciences, George Washington University School of Medicine and Health Sciences, Washington, DC; 6 ADHD (Attention-Deficit/Hyperactivity Disorder) Coaches Organization, ADHD Center of West Michigan, Grand Rapids; 7 Department of Pediatrics, University of Melbourne, Melbourne, Australia; 8 Department of Psychiatry, University of Melbourne, Melbourne, Australia; 9 Nova Southeastern University, Wayne Huizenga College of Business and Entrepreneurship, Fort Lauderdale, Florida; 10 ADHD Coaches Organization, San Diego, California; 11 Children and Adults with Attention Deficit/Hyperactivity Disorder, Lanham, Maryland

## Abstract

**Importance:**

Individuals with ADHD face great challenges accessing formal psychosocial treatment. ADHD coaching has rapidly emerged online as a popular and accessible form of grassroots psychosocial support; however, there is a paucity of documentation about this workforce or the safety and effectiveness of its services.

**Objective:**

To document patterns in the ADHD coaching workforce, including workforce entry dates, services, and coach characteristics.

**Design, Setting, and Participants:**

The US National Survey on ADHD Coaching was conducted from October 1, 2024, to April 3, 2025, using a purposive electronic survey and snowball sampling. The survey was distributed online and in person to ADHD community hubs, professional listservs, and direct outreach to self-identified ADHD coaches providing care to at least 1 client in the US during the past year.

**Main Outcomes and Measures:**

The ADHD coaching workforce’s characteristics and practices were assessed using descriptive statistics.

**Results:**

The survey was completed by 481 ADHD coaches. Among 464 coaches who provided age data, the mean (SD) age was 51.3 (11.5) years. Among 467 coaches who provided gender data, 381 (81.6%) identified as women. Most of the ADHD coaching workforce (283 of 465 [60.9%]) began practicing during or after the COVID-19 pandemic, actively received referrals from health care professionals (312 of 481 [64.9%]), and served clients across state lines (389 of 481 [80.9%]). Many practiced internationally (194 [40.3%]). Most participants self-identified as either having or suspecting they have ADHD (338 of 465 [72.7%]), used lived experience with ADHD to inform their services (460 of 465 [98.9%]), and previously received ADHD coaching (207 of 465 [44.5%]). Coaches were typically self-employed (440 of 481 [91.5%]), worked from home (417 of 480 [86.9%]), had no professional license (396 of 466 [85.0%]), and operated without formal clinical supports (420 of 464 [90.5%]). ADHD coaches predominantly offered virtual 1:1 weekly sessions with self-pay fees similar to those of psychotherapists, advertising via social media or other online platforms. While 417 of 467 (89.3%) reported no professional background in mental health, 292 of 467 (62.5%) completed an ADHD coach–led curriculum prior to workforce entry. Most ADHD coaches reported using executive function skills training, cognitive restructuring, and motivational interviewing. They discussed clinical topics such as mental health crises, substance use, trauma, and medication adherence.

**Conclusions and Relevance:**

The findings of this survey study suggest that there was a spike in ADHD coaching workforce entry at the outset of the COVID-19 pandemic that continues presently. Randomized clinical trials are essential to establish the safety and effectiveness of ADHD coaching. Conditional on positive findings of these trials, the health care and ADHD coaching communities might collaborate to develop standardized educational, credentialing and/or licensing, and clinical oversight.

## Key Points

**Question:**

What are the workforce characteristics and common practices of the rapidly expanding attention-deficit/hyperactivity disorder (ADHD) coaching sector?

**Findings:**

In this survey study including 481 participants in the US, ADHD coaching was documented as a grassroots, online form of support offered by lay individuals reporting lived experience with ADHD, with a spike in ADHD coaching workforce entry appearing since the COVID-19 pandemic. ADHD coaches largely operate outside the traditional health care system, providing services in executive function skills, adaptive thinking, and motivational enhancement.

**Meaning:**

These findings suggest that ADHD coaching is largely practiced by lay people with ADHD lived experience and that standards, effectiveness, safety, and appropriate regulatory safeguards must be established.

## Teaser

This survey study investigates the attention-deficit/hyperactivity disorder (ADHD) coaching workforce in the US and examines its business practices, pathways to coaching, demographic characteristics, coaching background, coaching style, and interprofessional connections.

## Introduction

Attention-deficit/hyperactivity disorder (ADHD) is a lifespan neurodevelopmental disorder that, when untreated, serves as a precursor to serious outcomes, including depression, suicide, addiction, accidents, unemployment, divorce, abuse perpetration and/or exposure, illness, and even premature death.^1,2^ Treatment guidelines recommend multipronged interventions targeting core symptoms through the brain’s neurochemical environment (ie, stimulant and/or nonstimulant medications) and impairments through behavioral or cognitive-behavioral therapy (CBT) targeting self-regulation, lifestyle changes, psychological health, and person-environment fit.^3,4^

The US faces an unprecedented supply-demand imbalance in ADHD treatment, with nearly 40% of affected individuals not receiving treatment (medication or psychosocial).^5^ This gap is exemplified by the well-documented stimulant shortage.^5^ Demand for ADHD care spiked since 2021 due to a wave of new diagnoses,^5,6,7,8^ likely stemming from several trends (eg, telehealth increasing care access, relaxed diagnosis by health care practitioners, compounded work-life demands and/or stressors, digital distractors, direct-to-consumer marketing by telehealth startups, #ADHD social media content, and increased self-diagnosis).^9^ Outsized demand for pharmacological care was met by an expanding prescriber workforce.^10^ Increased demand for psychosocial treatment appears to have been met outside health care systems by online ADHD coaching. In 2024, nearly 1 in 5 adults and 1 in 7 children with ADHD received ADHD coaching.^11^

ADHD coaching is a grassroots form of psychosocial care that emerged from the life coaching movement^12^ but is unique in its focus on executive functioning, self-regulation, and behavioral scaffolding for ADHD.^13^ Coaching is defined as a relationship between a coach and client aimed at attaining professional or personal goals. Originating in corporate settings in the 1990s, this unregulated support movement has since expanded into all potential domains of life.^12^ ADHD coaches tend to operate outside traditional health care systems without formal clinical supports, mental health training, or credentialing from regulatory bodies.^14^ ADHD coaches describe their field as informed by best practices in psychosocial ADHD treatment^13^ but with a neurodiversity-affirming reframe that emphasizes the coach’s own lived experiences with ADHD.^14^ However, research on ADHD coaching is slim, with just a few case studies, small pretreatment-posttreatment evaluations, and 1 randomized clinical trial (RCT) helping college students with study skills.^15,16,17,18^

Presently, there are many unanswered questions about characteristics of the ADHD coaching workforce, its practices, and the safety and effectiveness of its services. There are several successful examples of how incorporating grassroots providers can close critical US health care gaps through workforce expansion or modification of traditional health care models (eg, substance use peer counselors emerged from community support models such as Alcoholics Anonymous; military medics returned stateside to become the first US physician assistants).^19,20^ Perhaps ADHD coaches, who have their own practice organization (ADHD Coaches Organization [ACO]), might do the same for ADHD psychosocial care.^21^ However, the effectiveness of ADHD coaching and whether it aligns with best practices in ADHD intervention (eg, using evidence-based components and measurement-based care) is unknown. The safety of ADHD coaching also remains untested with questions about potential spread of misinformation about ADHD to clients,^22^ reinforcing inaccurate self-diagnoses, and management of clinical concerns without oversight.^23^ It is unknown whether some ADHD coaches may inadvertently engage in suboptimal intervention processes (eg, harmful advice-giving, inappropriate disclosures, projection, countertransference),^24^ which might require coordinated deimplementation. These concerns are particularly heightened as ADHD coaches reportedly work with vulnerable child and adolescent populations.^11^

Despite ADHD coaching’s still emerging data, it is now recommended in several ADHD practice guidelines,^3,25^ and patients are reporting higher satisfaction with ADHD coaching than traditional CBT.^11^ Patient preference for ADHD coaching over CBT may be compounded by a mental health therapy sector that deprioritizes ADHD over other diagnoses^26,27^ and can be unempathetic to the plight of clients with ADHD who may arrive late to sessions, inconsistently follow through on intentions, or struggle to connect interpersonally.^28^ ADHD coaches, in contrast, appear very interested in devoting their professional career to helping people with ADHD thrive, but may need training, oversight, structures, and supports to do so effectively.^14^

To summarize, the characteristics and practices of ADHD coaches are largely unknown, despite the rapid expansion of this care in the US. Therefore, we conducted the US National Survey on ADHD Coaching, which aims to document the (1) coaching business practices, (2) pathways to ADHD coaching, (3) demographic characteristics and coaching background, (4) coaching style, and (5) interprofessional connections of the US ADHD coaching workforce. This information may be used to inform ADHD research, practice, policy, and guidelines.

## Methods

The US National Survey on ADHD Coaching followed the American Association for Public Opinion Research (AAPOR) reporting guideline for survey research. The University of Washington Institutional Review Board approved the research. All procedures complied with ethical guidelines for human participant research. Participants provided informed consent electronically before completing the anonymous, online survey. This study was preregistered with OSF.^29^

### Participants and Procedures

Participants included 481 self-identified ADHD coaches who completed the 60-item English-language survey from October 1, 2024, to April 3, 2025. We included ADHD coaches who provided services to clients located in the US, a US territory, or a US military base and had done so in the last 12 months. Because this workforce has never been systematically surveyed or documented, we recruited participants using a multimodal outreach strategy with purposive and snowball sampling. Our recruitment process included emailing professional listservs and social media postings by organizations likely to include ADHD coaches (ie, the ADHD Coaches Organization, Children and Adults with Attention-Deficit/Hyperactivity Disorder [CHADD], the International Coaching Federation, and alumni lists from ADHD coaching courses) and ADHD coaching businesses, recruiting in person at ADHD conferences, sharing content on branded social media accounts, and using ADHD community platforms (eg, *ADDitude Magazine*, CHADD’s online support site). We also identified ADHD coaches through online searches of ADHD coaching personal and/or professional websites, ADHD coaching directories, social media accounts, and Google Maps. We sent emails or direct messages to identified individuals inviting them to visit the study website for more information. We encouraged ADHD coaches to share recruitment information with professional networks.

For survey distribution, we used a 2-step process. First, participants requested a survey link through a form embedded in the study website. Second, they then received an anonymous Qualtrics survey link to the provided email.

### Data Integrity

To protect against fraudulent responses, we implemented data integrity measures consistent with best practices for survey security and bot prevention and/or detection.^30,31,32,33,34,35,36^ We did not provide compensation and implemented a 2-step authentication process for survey access. We enabled several Qualtrics features, including ReCAPTCHA fraud detection, RelevantID duplicate detection, Security Scan Monitor, and Prevent Indexing. During manual data review, we confirmed that no participants completed the survey in less than 5 minutes, removed all responses identified by Qualtrics as potential bots (n = 2), and verified and removed duplicate responses (n = 31).

### Measures

The US National Survey on ADHD Coaching was an online survey with 50 quantitative and 10 qualitative questions divided into 5 subcategories: coaching practice, pathway to ADHD coaching, demographic characteristics and coaching background, coaching style, and interprofessional connections. Race and ethnicity data were based on ADHD coaches’ self-report and were assessed to understand how the sample of ADHD coaches reflects the diversity of clients served; categories included African American or Black, American Indian or Alaska Native, Asian or Pacific Islander, Hispanic, non-Hispanic White, and other race or ethnicity (including Ashkenazi Jewish, Black, Hispanic White, Indian or South Asian, Latino, White, and preferred not to say). The survey also had an optional qualitative component assessing coaches’ views and perspectives. Survey development included meetings and solicitation of questions from the ADHD Coaches Organization leadership, as well as formation of an advisory committee consisting of coaches, researchers, clinicians, individuals with ADHD, and other stakeholders. Survey refinement occurred through iterative feedback with the advisory committee. Prior to launch, a pilot sample of 6 ADHD coaches took the survey to report on completion time and issues with survey functionality.

### Statistical Analysis

We summarized ADHD coaches’ responses descriptively across key demographic, service delivery, and practice characteristics. We compiled summary statistics including frequencies and percentages for categorical variables, means and SDs for normally distributed variables, and medians and ranges for nonnormally distributed variables. Of 792 unique requests to take the survey, 670 respondents accessed the survey, and 514 initially self-reported that they met study eligibility and consented. After removing duplicates and potential bots, 481 ADHD coaches were included in our analyses. Missingness was low (<5%) across all study variables, and given the descriptive approach to analyses, no imputation was conducted.

## Results

**Figure 1.**
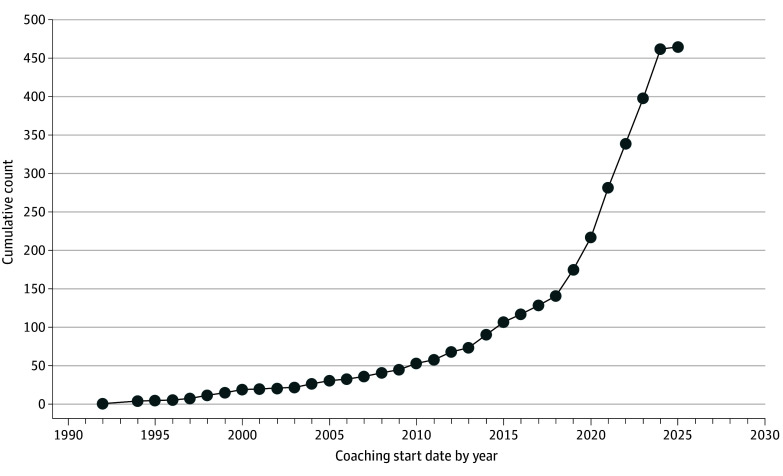
Coaches Entering Into the Attention-Deficit/Hyperactivity Disorder Coaching Workforce by Year Survey data collection ceased in April 2025; 2025 data represent a partial figure of the likely workforce entry in 2025.

**Figure 2.**
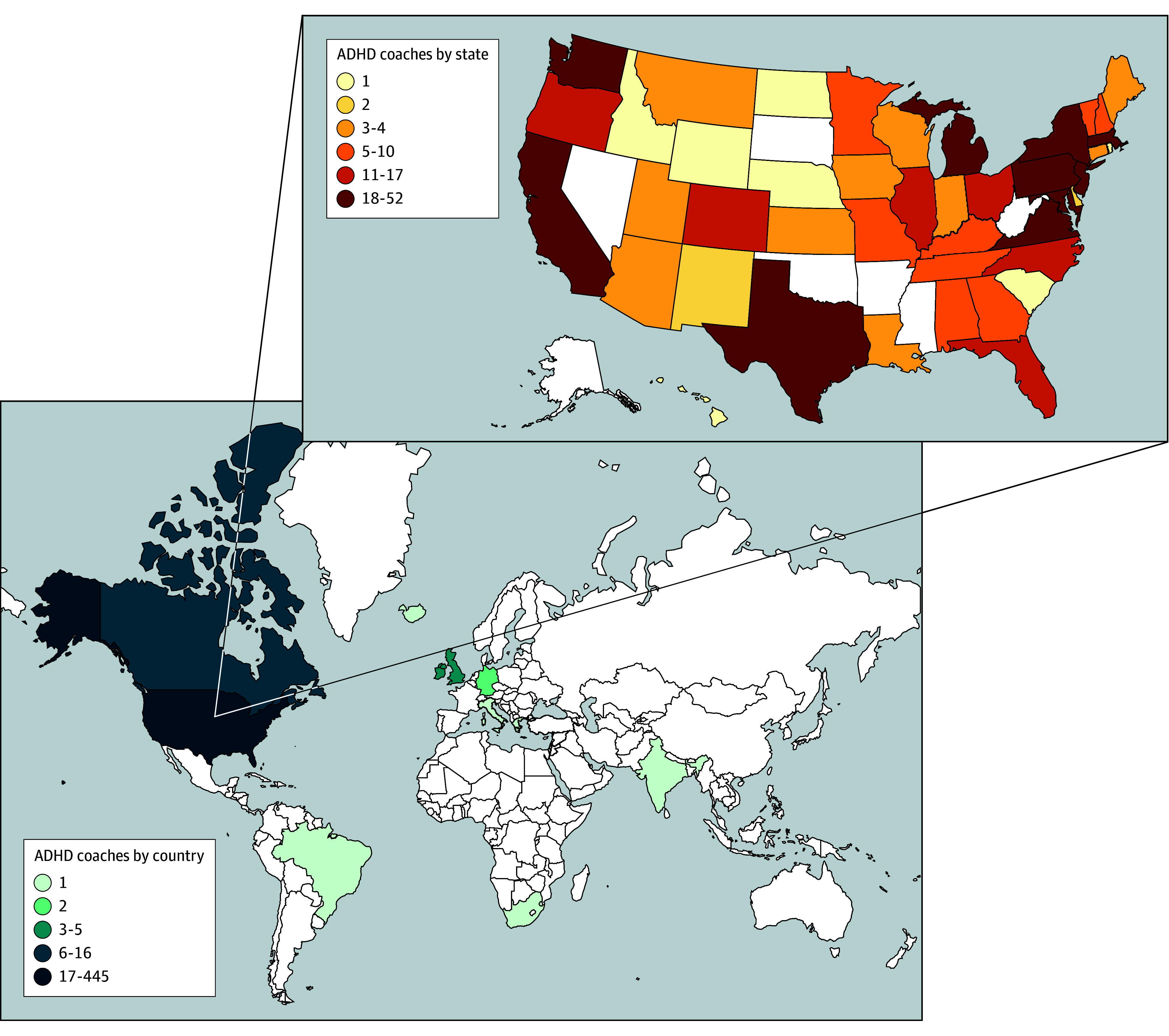
Sampled Attention-Deficit/Hyperactivity Disorder (ADHD) Coaches by State and Country Count of coaches represents data derived from self-reported zip code or country of residence if not within the US. The eFigure in Supplement 1 provides the frequency of coaches per state that is adjusted to account for the population proportions by state.

Demographic characteristics of the 481 ADHD coaches are summarized in Table 1. As demonstrated in Figure 1, our survey substantiated reports of a rapidly expanding ADHD coaching workforce. Over half the sample (283 of 465 [60.9%]) entered the ADHD coaching field during or after the COVID-19 pandemic. Figure 2 and the eFigure in Supplement 1 demonstrate geographic locations of participating ADHD coaches.

**Table 1.** ADHD Coach Characteristics^a^

Characteristic	No./total No. (%) of participants
Lived experiences	
Self-identify as having or suspecting they have ADHD	338/465 (72.7)
Received formal ADHD diagnosis^b^	274/465 (58.9)
Received ADHD coaching as client^b^	207/465 (44.5)
Family member with ADHD	361/465 (77.6)
Friends or romantic partner with ADHD	280/465 (60.2)
Teaching or working professionally with individuals with ADHD	310/465 (66.7)
No lived experience with ADHD	5/465 (1.1)
Gender	
Men	74/467 (15.8)
Women	381/467 (81.6)
Nonbinary, gender queer, transgender, or other	23/467 (4.9)
Age, y^c^	
≤39	78/464 (16.8)
40-49	121/464 (26.1)
50-59	158/464 (34.1)
≥60	107/464 (23.1)
Race and ethnicity	
African American or Black	11/467 (2.4)
American Indian or Alaska Native	5/467 (1.1)
Asian or Pacific Islander	16/467 (3.4)
Hispanic	21/467 (4.5)
Non-Hispanic White	416/467 (89.1)
Other^d^	15/467 (3.2)
Educational level, degree attained^c^	
Less than bachelor’s	13/467 (2.8)
Bachelor’s	134/467 (28.7)
Master’s	253/467 (54.2)
Doctoral	56/467 (12.0)
Health care licensure	
Yes	70/466 (15.0)
No	396/466 (85.0)
Job sector prior to coaching^c^	
Education	147/467 (31.5)
Business	51/467 (10.9)
Mental health	50/467 (10.7)
Other	184/467 (39.4)
None	35/467 (7.5)
Professional engagement^c^	
Completed an ACO-endorsed ADHD coach-led training curriculum^e^	292/467 (62.5)
Member of a professional ADHD coaching organization^f^	271/467 (58.0)

Abbreviations: ACO, ADHD Coaching Organization; ADHD, attention-deficit/hyperactivity disorder.

^a^ Unless otherwise indicated, responses were not mutually exclusive, and respondents could select all that apply.

^b^ Items were only asked to those who self-identify as having ADHD.

^c^ Category is mutually exclusive.

^d^ Includes Ashkenazi Jewish, Black, Hispanic White, Indian or South Asian, Latino, White, and preferred not to say.

^e^ Includes individuals who endorsed completing one of the ADHD coaching training courses listed on the ACO website.

^f^ Includes membership in the ACO or the Professional Association of ADHD Coaches.

### Coach Demographic Characteristics

Most ADHD coaches self-identified as either having or suspecting they have ADHD (338 of 465 [72.7%]); 207 of 465 (44.5%) had received ADHD coaching as a client. Prior to ADHD coaching, 460 of 465 (98.9%) reported significant lived experiences with ADHD, including a close family member with ADHD or previously holding a profession serving individuals with ADHD (eg, special education teacher). Among 464 coaches who provided age data, the mean (SD) age was 51.3 (11.5) years. Among 467 coaches who provided gender data, 74 coaches (15.8%) identified as men, 381 (81.6%) as women, and 23 (4.9%) as nonbinary, gender queer, transgender, or other gender. In terms of race and ethnicity, 11 coaches (2.4%) identified as African American or Black, 5 (1.1%) as American Indian or Alaska Native, 16 (3.4%) as Asian or Pacific Islander, 21 (4.5%) as Hispanic, 416 (89.1%) as non-Hispanic White, and 15 (3.2%) as other race or ethnicity. Most participants held at least a bachelor’s degree (443 of 467 [94.9%]); many held advanced degrees (309 of 467 [66.2%]), but primarily in fields unrelated to mental health. Most coaches had no health care licensure (396 of 466 [85.0%]) or background in mental health (417 of 467 [89.3%]) and entered the profession after a career change (432 of 467 [92.5%]). The most common previous sector was education (147 of 467 [31.5%]). More than half of ADHD coaches completed an ADHD coach-led training curriculum endorsed by the ACO (292 of 467 [62.5%]) and were members of an ADHD coaching professional organization (271 of 467 [58.0%]).

### Service Delivery

Most ADHD coaching delivered to US clients originated domestically (445 of 481 [92.5%]). Among all 481 participants, 389 (80.9%) practiced across state lines and 194 (40.3%) across international borders (Table 2). The primary modality offered was individual sessions (472 of 480 [98.3%]); most delivered services virtually (467 of 481 [97.1%]) and from their homes (417 of 480 [86.9%]). Most coaches (398 of 481 [82.7%]) reported providing client support between sessions, such as encouragement, reminders to complete tasks, and prompts using digital technology. Most coaches (440 of 481 [91.5%]) were self-employed or were operating independently, and 297 of 481 (61.7%) reported professional liability insurance. Most reported marketing their services using online directories (343 of 481 [71.3%]) and/or social media platforms (234 of 481 [48.6%]) or receiving direct referrals from clinicians (312 of 481 [64.9%]). Most coaches (429 of 481 [89.2%]) reported mainly serving adults who were not college students. A substantial number also saw children (99 of 481 [20.6%]) and adolescents (217 of 481 [45.1%]). Many coaches (301 of 481 [62.6%]) reported that most of their clients had at least a bachelor’s degree; most reported that a majority of their clients were White (333 of 481 [69.3%]) and English speakers (458 of 481 [96.4%]). Most coaches (444 of 481 [92.3%]) perceived that most of their clients are receiving ADHD coaching as an adjunct to formal ADHD treatments. Approximately 90% of clients paid out-of-pocket for services. The median frequency of sessions was weekly, with a mean (SD) session duration of approximately 53.9 (10.6) minutes, and median care duration of 6 (range, 1-75) months.

**Table 2.** Characteristics of ADHD Coaching Services^a^

Characteristic	No./total No. (%) of participants
Coach lives in the US	445/481 (92.5)
Coaching delivery	
Across state lines	389/481 (80.9)
Across international borders	194/481 (40.3)
Coaching modality	
Virtual	467/481 (97.1)
In-person	212/481 (44.1)
Provide between session support	398/481 (82.7)
Coaching format	
Individual session	472/480 (98.3)
Family session (eg, couples, parents)	167/480 (34.8)
Group session	141/480 (29.4)
Live seminar or workshop	92/480 (19.2)
Prerecorded content	38/480 (7.9)
Text-based coaching	2/480 (0.4)
Location of coach during sessions	
Coach’s home	417/480 (86.9)
Private professional space	163/480 (34.0)
Public location (eg, coffee shop, park, library)	59/480 (12.3)
Client’s home	50/480 (10.4)
Coaching practice	
Self-employed	440/481 (91.5)
Work for a coaching practice	46/481 (9.6)
Work for a health care practice	17/481 (3.5)
Work for an educational institution	19/481 (4.0)
Other	3/481 (0.6)
Payment and/or funding accepted	
Self-pay	458/481 (95.2)
Pro bono	130/481 (27.0)
Flexible spending or health savings account	130/481 (27.0)
Client’s employer	109/481 (22.7)
Health insurance	21/481 (4.4)
Professional liability insurance coverage^b^	
Yes	297/481 (61.7)
No	184/481 (38.3)
Source of clients	
Advertisement in online directory	343/481 (71.3)
Referrals from clinicians	312/481 (64.9)
Social media marketing	234/481 (48.6)
Referrals from educational sector	135/481 (28.1)
Paid advertisements	98/481 (20.4)
Word of mouth	88/481 (18.3)
Other	135/481 (28.1)
Population served	
Adults (nonstudents)	429/481 (89.2)
College students	324/481 (67.4)
Adolescents (aged 12-17 y)	217/481 (45.1)
Children (aged <12 y)	99/481 (20.6)
Primarily individuals who receive formal ADHD services	444/481 (92.3)
Primarily White individuals	333/481 (69.3)
Primarily women or girls	145/481 (30.1)
Primarily adults with at least a bachelor’s degree	301/481 (62.6)
Entirely English language speakers	458/481 (96.4)
No. of clients on caseload, median (range)	10 (0-300)
Length of sessions, median (range), min	55 (20-120)
No. of sessions per month per client, median (range)	4 (1-160)
Duration of active services per client, median (range), wk^c^	24 (4-300)
Total weekly direct client hours, median (range)	8 (0-60)
Total weekly indirect hours, median (range)	5 (0-102)
Individual session rate or fee, median (range), USD^d^	$150 ($0-$750)
Self-paying cases, median (range), %^e^	100 (1-100)

Abbreviation: ADHD, attention-deficit/hyperactivity disorder.

^a^ Unless otherwise indicated, responses were not mutually exclusive, and respondents could select all that apply.

^b^ Category is mutually exclusive.

^c^ Includes 478 participants.

^d^ A total of 470 participants reported a dollar amount for their individual session rate or fees and 2 reported fees of $3000 or more that were winsorized to the next highest value ($750) to reduce the impact of these outliers in positively skewing the data.

^e^ Includes 457 participants.

### ADHD Coaching Practices

Most ADHD coaches (420 of 464 [90.5%]) were not receiving clinical supervision. However, 304 of 464 coaches (65.5%) reported receiving peer consultation from another ADHD coach and 149 of 464 (32.1%) sought informal consultation from clinicians. During ADHD coaching sessions, most coaches report conceptualizing ADHD as a form of neurodiversity (438 of 465 [94.2%]), using no structured materials or using materials they developed themselves (342 of 464 [73.7%]), and using practices that largely mirror best practices in adult ADHD psychosocial treatment, for example, executive function skills training or targeting self-motivation (461 of 464 [99.4%]), cognitive restructuring (461 of 464 [99.4%]), motivational interviewing (448 of 464 [96.6%]), and solution-focused approaches (454 of 464 [97.8%]). Most (420 of 464 [90.5%]) also reported sharing their own lived experiences with ADHD during sessions. ADHD coaches reported regularly discussing a range of clinical concerns with clients (eg, sleep management, emotional concerns, ADHD medication adherence, substance use and/or addictions, trauma, suicidality) (Table 3). All queried practices in measurement-based care were reportedly used by less than half of ADHD coaches (Table 3). ADHD coaches frequently reported referring clients to clinicians, particularly for formal ADHD evaluation (366 of 464 [78.9%]) and medication treatment (283 of 464 [61.0%]), and somewhat less frequently, to CBT (236 of 464 [50.9%]).

**Table 3.** ADHD Coaching Practices

Practice^a^	Frequency, No./total No. (%) of participants	Mean (SD) response^b^
Receipt of supervision or informal consultation		
Peer consultation from another coach	304/464 (65.5)	NA
Informal consultation from a licensed clinician	149/464 (32.1)	
Clinical supervision from a licensed clinician	44/464 (9.5)	
Conceptualization of ADHD (n = 465)		
Form of neurodiversity	438/465 (94.2)	NA
Neurodevelopmental disorder	222/465 (47.7)	
Mental health condition	94/465 (20.2)	
Psychiatric disorder	35/465 (7.5)	
Mental disorder	28/465 (6.0)	
Structured intervention materials used		
Curriculum or protocol developed by someone else^c^	122/464 (26.3)	NA
Curriculum or protocol developed by self	152/464 (32.8)	
None	190/464 (40.9)	
Intervention approaches used		
Executive function skills training and/or self-motivation strategies	461/464 (99.4)	1.83 (0.40)
Cognitive restructuring	461/464 (99.4)	1.83 (0.40)
Solution-focused approach	454/464 (97.8)	1.68 (0.51)
Motivational Interviewing	448/464 (96.6)	1.60 (0.56)
Sharing own lived experience	420/464 (90.5)	1.12 (0.54)
Between session homework assignments	385/464 (83.0)	1.11 (0.67)
Clinical concerns: topics covered		
Sleep	456/464 (98.3)	1.52 (0.53)
Self-worth	455/464 (98.1)	1.68 (0.51)
Emotional concerns	450/464 (97.0)	1.48 (0.56)
Health behavior (eg, nutrition, exercise, managing medical conditions)	450/464 (97.0)	1.38 (0.54)
Parenting	378/464 (81.5)	1.08 (0.67)
ADHD medication therapy adherence	361/464 (77.8)	0.99 (0.66)
Substance use or addictions	246/464 (53.0)	0.59 (0.60)
Trauma	226/464 (48.7)	0.56 (0.63)
Suicide, abuse, and/or harm to self or others	193/464 (41.6)	0.47 (0.59)
Use of measurement-based care		
Progress monitoring	208/464 (44.8)	NA
Interpret outside evaluations	158/464 (34.1)	
No assessment	142/464 (30.6)	
Collect client satisfaction ratings	136/464 (29.3)	
Conducts ADHD diagnostic assessments	56/464 (12.1)	
Outgoing referrals to health care		
A (mental) health care clinician to obtain formal diagnosis	366/464 (78.9)	NA
Medication prescribers	283/464 (61.0)	
CBT clinicians	236/464 (50.9)	
Holistic, naturopathic, or other healing arts professionals	99/4v64 (21.3)	

Abbreviations: ADHD, attention-deficit/hyperactivity disorder; CBT, cognitive behavioral therapy; NA, not applicable.

^a^ All ADHD coaches were asked all coaching practice questions, regardless of the age group they reported serving in their caseload.

^b^ Response choices ranged from none (0), some (1), and a lot (2). Responses of some and a lot were collapsed together to describe any use for the frequency or percentage.

^c^ Includes structured curricula, manuals, or programs developed by their employer (n = 114), or someone else (n = 8).

## Discussion

This survey study found that most ADHD coaches primarily operated outside the US health care system and reported workforce entry after the COVID-19 pandemic’s onset. Our findings suggest ADHD coaching is usually delivered through a 1:1 virtual format using a traditional outpatient psychotherapy model (weekly 1-hour sessions) and reached prospective clients through a combination of online marketing and health care referrals. ADHD coaches tended to be individuals without formal mental health training who self-identified as having ADHD (or a loved one with ADHD), may have received ADHD coaching themselves, and based practices on lived experiences. Unlike most licensed mental health clinicians, ADHD coaches practiced across state and international borders.

As expected, we detected a spike in ADHD coaching workforce entry at the COVID-19 pandemic’s outset that mirrored similar ADHD medication prescribing patterns.^6^ Herein, we reveal that intervention content self-reported by ADHD coaches is similar to those manualized in evidence-based CBTs for ADHD.^37^ The potential redundancy in content between ADHD coaching and CBT for ADHD could make it difficult for prospective clients and some medical clinicians to differentiate between these approaches. However, the aforementioned aspects of ADHD coaching are different than traditional CBTs in that ADHD coaching appears longer term, involves sharing lived experiences with ADHD, and offers support between sessions (Table 2).^38,39,40^ These features may make ADHD coaching especially palatable to adults with ADHD, who reportedly criticize routine care CBT as being too rigid, generic, and short term, with therapists who are stigmatizing, negativistic about ADHD, and unempathetic.^28,41^ If consumers find ADHD coaching more palatable than CBT, they may be more likely to initially engage and sustain participation.

On top of its consumer appeal, ADHD coaching also may offer an opportunity to improve the availability and quality of ADHD psychosocial care; most routine care psychotherapists do not list ADHD as a presenting problem they treat (especially for adults) (unpublished data; A. Gaddis, BA, M, Rout, BA, M.H.S., C.E.Y., N. Groves, PhD; December 2025) and are viewed by clients as insufficiently specialized in ADHD.^28^ In contrast, ADHD coaches advertise highly sought-after specialty psychosocial services for ADHD; however, they may be basing that specialization on lived experience rather than professional training. Importantly, ADHD coaching should not be viewed as equivalent to evidence-based psychotherapy for ADHD, as there are many potential drawbacks when pivoting to unsupervised, lay-practitioner treatment models. In addition to potential reduced efficacy if evidence-informed approaches are delivered inconsistently or incorrectly, adverse effects may include spread of misinformation about ADHD, risk of giving harmful advice, challenges maintaining professional boundaries, and ethical concerns such as loss of patient confidentiality. RCTs must establish efficacy and qualitative research with traditional health care professionals should explore existing clinician viewpoints on the value of ADHD coaching to their patients. The risk of adverse effects may be particularly important to monitor for children and adolescents receiving ADHD coaching, as ADHD coaches do not appear to have specialized training in child development. Children may be more vulnerable to long-term negative consequences of harmful therapeutic approaches, particularly as they may struggle to report negative experiences and may reflexively view adults as trustworthy authority figures.

### Implications

Previous research suggests that the community of patients with ADHD reports positive experiences with ADHD coaching and frequently engages in these services online.^11^ However, ADHD coaching appears to have infrequent clinical oversight from the health care sector to support clinical needs raised in sessions (Table 3). Although presently lay providers, ADHD coaches may one day follow the trajectory of other successful workforce expansion efforts (eg, peer substance use counselors, physician assistants) through (1) research validation using RCTs, (2) willingness of Centers for Medicare & Medicaid Services to reimburse services, (3) establishment of national education and credentialing standards by a well-recognized professional health care organization, and (4) state-level licensure. Until this professional formalization occurs, ADHD coaching will be vulnerable to systemic inequities,^11^ as its costs are currently largely out-of-pocket and ADHD coaching predominantly reaches White, college-educated, English-speaking US residents (and their children). If future work indicates a cost-benefit and/or cost-effectiveness of ADHD coaching, there also may be incentives for insurers and health care systems (particularly those engaged in value-based contracts) to offer these services.

Health care professionals should be aware that patients will commonly pursue ADHD coaching as a form of care. They should understand that, at first glance, ADHD coaching and CBT may appear interchangeable; however, there are key differences in who delivers the services and how they are delivered that may impact safety and effectiveness. Health care professionals may tolerate the use of ADHD coaching by clients, rather than discouraging it. However, they might educate patients on key considerations: (1) ADHD coaching is currently most appropriate as an adjunct rather than a sole form of care; (2) unlike CBT delivered by licensed mental health clinicians,^37,38,39,40^ ADHD coaching is still developing its evidence base, thus its likelihood of safely improving patient functioning is unknown; and (3) the absence of standards and regulations about who can declare themselves an ADHD coach make it particularly important to practice due diligence when vetting an ADHD coach, especially as there are no legal protections for clients.

### Limitations

This study has several limitations. As with all public survey research, our analyses are limited by unknown error. We also could not precisely calculate the response rate given our survey’s anonymous nature and insufficient information about whether individuals reached by our outreach strategy were eligible (eg, some may not have had active clients in the US). There is a risk of sampling or selection bias, as survey respondents may be more likely to engage in online activity (eg, following social media accounts, absorbing media in ADHD community hubs) or belong to professional organizations through which we advertised. They also may be more likely to demonstrate trust in researchers. This survey study relies on self-reported cross-sectional data, limiting the ability to assess temporal relationships about coaching practices, coaches, and the populations they serve. Some important research questions about ADHD coaching would be best answered by surveying clients, conducting RCTs, or using qualitative methodology (particularly questions of safety, effectiveness, and costs).

## Conclusions

This survey study detected a spike in ADHD coaching workforce entry at the outset of the COVID-19 pandemic that continues presently. In the US, ADHD coaching is largely delivered outside health care systems via online modalities and by individuals with ADHD lived experience who typically have no formal mental health training or oversight. Although ADHD coaching currently has a limited evidence base, health care practitioners appear to be regularly referring patients to ADHD coaching, and professional practice guidelines have begun recommending ADHD coaching.^3,25^ ADHD coaching demonstrates promise of scalability, but research is needed to clarify its safety, effectiveness, and potential cost savings. A public health response is warranted to ensure adequacy of care by this largely unregulated profession. Given that ADHD coaching is highly appealing to patients and has impressive scalability, partnership among the ADHD research, health care, and coaching communities could lead to a unique opportunity to improve the availability and costs of high-quality care to individuals with ADHD.

## Notes

Supplement 1. **eFigure.** Population-Adjusted Frequencies of ADHD Coach Residency by State

Supplement 2.
Data Sharing Statement
