# Supplementary material for: Demographics, Services, and Practices in Attention-Deficit/Hyperactivity Disorder Coaching in the US
Source: JAMA Netw Open. 2026 Jan 15;9(1):e2552407. doi: 10.1001/jamanetworkopen.2025.52407 (PMC12809363; doi:10.1001/jamanetworkopen.2025.52407)
Supplement: Supplement 1. — eFigure. Population-Adjusted Frequencies of ADHD Coach Residency by State [file jamanetwopen-e2552407-s001.pdf]

Supplementary Online Content

Sibley MH, Graham ED, Holbrook J, et al. Demographics, services, and practices in attention-deficit/hyperactivity disorder coaching in the US. *JAMA Netw Open.* 2026;9(1):e2552407. doi:10.1001/jamanetworkopen.2025.52407

eFigure. Population-Adjusted Frequencies of ADHD Coach Residency by State

This supplementary material has been provided by the authors to give readers additional information about their work.

© 2026 Sibley MH et al. *JAMA Network Open*.

eFigure. Population-Adjusted Frequencies of ADHD Coach Residency by State

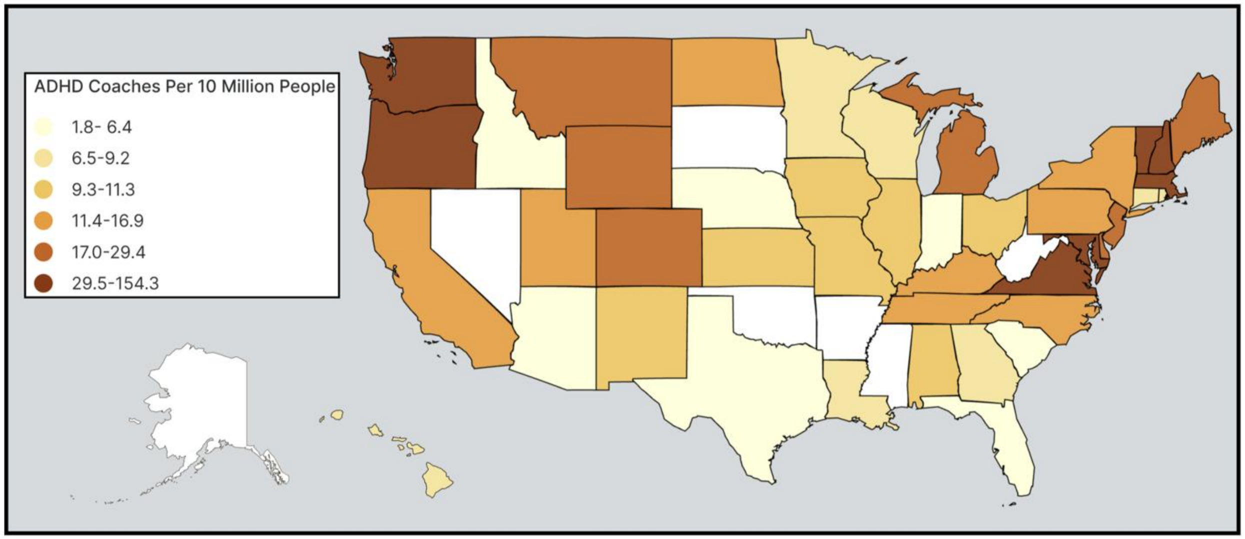

Note. Count of coaches represents data derived from self-reported zip codes. Frequency of coaches per state is adjusted to account for the population proportions by state.

© 2026 Sibley MH et al. *JAMA Network Open*.
