# Supplementary material for: Demographics, Services, and Practices in Attention-Deficit/Hyperactivity Disorder Coaching in the US
Source: JAMA Netw Open. 2026 Jan 15;9(1):e2552407. doi: 10.1001/jamanetworkopen.2025.52407 (PMC12809363; doi:10.1001/jamanetworkopen.2025.52407)
Supplement: Supplement 2. — Data Sharing Statement [file jamanetwopen-e2552407-s002.pdf]

# Data Sharing Statement

Sibley. Demographics, Services, and Practices in Attention-Deficit/Hyperactivity Disorder Coaching in the US. *JAMA Netw Open*. Published January 15, 2026.
doi:10.1001/jamanetworkopen.2025.52407

**Data**
**Data available:** Yes
**Data types:** Deidentified participant data, Data dictionary
**How to access data:** After the publication of this main outcome paper, we will be making all data publicly available on Open Science Framework at the following location: https://osf.io/mqgpf.
**When available:** With publication

**Supporting Documents**
**Document types:** Other (please specify)
**Additional Information:** The survey is made available in our protocol registration on OSF so that any other country that wishes to replicate this work can do so. We already have replication completed in Australia (currently in analysis stage) and underway in the UK.
**How to access documents:** https://osf.io/mqgpf
**When available:** beginning date: 09-01-2024

**Additional Information**
**Who can access the data:** This is already available.
**Types of analyses:** Any purpose.
**Mechanisms of data availability:** Without investigator support.
